# Estradiol valerate and alcohol intake: dose-response assessments

**DOI:** 10.1186/1471-2210-7-3

**Published:** 2007-03-04

**Authors:** Gina L Quirarte, Larry D Reid, I Sofía Ledesma de la Teja, Meta L Reid, Marco A Sánchez, Arnulfo Díaz-Trujillo, Azucena Aguilar-Vazquez, Roberto A Prado-Alcalá

**Affiliations:** 1 Departamento de Neurobiología Conductual y Cognitiva, Instituto de Neurobiología, Universidad Nacional Autónoma de México, Campus Juriquilla, Querétaro, 76230, México; 2 Labatory for Psychopharmacology, Rensselear Polytechnic Institute, Troy, NY, 12180 USA; 3 Universidad Autónoma de Querétaro, Querétaro, México; 4 Albany Public Library, Albany, NY, USA

## Abstract

**Background:**

An injection of estradiol valerate (EV) provides estradiol for a prolonged period. Recent research indicates that a single 2.0 mg injection of EV modifies a female rat's appetite for alcoholic beverages. This research extends the initial research by assessing 8 doses of EV (from .001 to 2.0 mg/female rat), as well assessing the effects of 2.0 mg EV in females with ovariectomies.

**Results:**

With the administration of EV, there was a dose-related loss of bodyweight reaching the maximum loss, when it occurred, at about 4 days after injections. Subsequently, rats returned to gaining weight regularly. Of the doses tested, only the 2.0 mg dose produced a consistent increase in intake of ethanol during the time previous research indicated that the rats would show enhanced intakes. There was, however, a dose-related trend for smaller doses to enhance intakes. Rats with ovariectomies showed a similar pattern of effects, to intact rats, with the 2 mg dose. After extensive histories of intake of alcohol, both placebo and EV-treated females had estradiol levels below the average measured in females without a history of alcohol-intake.

**Conclusion:**

The data support the conclusion that pharmacological doses of estradiol can produce enduring changes that are manifest as an enhanced appetite for alcoholic beverages. The effect can occur among females without ovaries.

## Background

Large doses of estradiol valerate (EV) deliver estradiol for 2 to 4 weeks. The released estradiol will have the same effects as estradiol delivered by other means; except, of course, for effects that accrue from the delivery of supraphysiological doses for an extended period [1]. A single large dose of EV (i.e., 2 mg/female rat) is reported to be toxic to the arcuate nucleus of the hypothalamus, thereby disrupting production of β-endorphin [2-4].

In the interest of determining the effects of reduced β-endorphin on appetite for alcoholic beverages, Reid et al. [5] provided female rats a daily opportunity to take sweetened alcoholic beverage. After the females had developed the habit of taking considerable alcohol daily, they were given EV (2 mg). The initial effect was a reduction in intake of alcohol, a finding concordant with the observations of others [6,7] and with some theory of the relationship of opioids to appetite for alcoholic beverages [8]. With the continuance of the daily opportunities to take alcoholic beverages, however, a different picture emerged. The EV-treated females gradually increased their intakes until they were taking more alcohol than the placebo-treated. Further, the enhanced intakes, once they emerged, were sustained for months after the single injection of EV [5,9].

Many events will reduce rats' intakes of alcoholic beverages, e.g., those associated with illness or a malaise. Only a few circumstances enhance appetite for ethanol for a sustained period. Given that an enhanced appetite for ethanol is the prime circumstance of alcohol abuse and alcoholism (AAA), the fact that a hormonal manipulation led to large, sustained intakes of an alcoholic beverage motivated further investigation. Further research [5,9,10] determined that the enhanced intake of ethanol was not specific to a particular strain of rats and not specific to a particular alcoholic beverage (i.e., increased intakes occurred with both sweetened and unsweetened alcoholic beverages and with different concentrations of ethanol). The increases in intake of ethanol were seen when alcoholic beverages were presented for 2 hr a day or when presented for 24 hr a day. The grams of ethanol per kilogram of body weight (g'E/kg) taken by EV-treated female rats can reach very large amounts. For example, when EV-treated females were presented with a saccharin-sweetened alcoholic beverage, for 24 hr a day from 1 to 3 months after a single injection of 2.0 mg of EV, 40% of them took over 8 g'E/kg a day, with the balance taking considerable amounts as well [9].

Are the enhanced appetites that emerge following a dose of EV specific to alcoholic beverages or might they occur with any beverage taken daily? To get germane information, female rats were given a daily opportunity to take saccharin solutions [11]. One group was provided with a solution taken avidly and one was provided with a concentrated solution that was taken only modestly (to the human taste, it is bitter sweet). Half of the females were given the same dose of EV that enhanced intakes of alcoholic beverages and the other half were given placebos. The single dose of EV enhanced intakes of the more palatable saccharin solution, but decreased intakes of the less palatable solution. The conclusion is that EV can enhance intakes of highly palatable, reinforcing ingesta, but not intakes of all ingesta.

Following a single injection of EV, 2.0 mg/rat, there is weight-loss across the first few days. Then, there is a resumption of weight-gain with nearly the same rate of gain as placebo controls [5,9,11]. The changes in weight are interesting, because they probably reflect underlying conditions that affect appetite. The initial loss of weight may index a malaise that could be conditioned to presented ingesta. If a malaise is a possibility with injections of estradiol (as indexed by weight loss), then the conclusion that estradiol reduces intake of alcoholic beverages [6,7] and intake of other ingesta [12] may be related to a conditioned food avoidance [13,14]. As the malaise and consequent food avoidance wanes, however, the continuance of estradiol enhances intake of alcoholic beverages.

As mentioned, many events can reduce rats' intakes of alcohol (e.g., a malaise), but only a few events, programmed in the laboratory, increase rats' intakes. One reliable variable for increasing alcohol-intake is extended opportunities to take alcoholic beverages. Another reliable variable, for increasing intake, is to make the alcoholic beverage palatable (e.g., by adding sweeteners [15] or the taste of beer [16]). Yet another variable that will increase intakes is to deprive rats of food or water before opportunity to take food and water as well as alcoholic beverage[17]. The hunger and thirst seem to set a condition that increases the ingestion of alcoholic beverage even though intake of food or water is the first priority. Similarly, rats take more alcoholic beverage when presented during the time they ordinarily eat and drink the most (during the first part of the dark cycle) compared to the same duration at other times. After sleep, they are usually food and water deprived for a number of hours. During the initial arousal of their day, they drink, eat, groom and, if alcoholic beverages are available, take the [18]. Female rats, on average in terms of g'E/kg, take more ethanol than the males of the same strain [19-21]. As will be demonstrated here, these variables can be combined (present a sweetened alcoholic beverage for a number of days at the time when female rats usually eat and drink) to induce large intakes of ethanol. Will doses of EV, other than those used initially, induce further increases in intakes? Only two doses of EV have been assessed, 2.0 and 1.0 mg per rat [5,9,10]. A purpose of this study is to test more doses. If smaller doses increase intakes, then it is more likely that the doses of estradiol used by women might enhance appetite for alcoholic beverages.

There is theory indicating that the toxicity associated with large doses of EV is a product of an interaction with the ovaries [22]. In light of those ideas, we performed ovariectomies on one group before getting their EV (2 mg) and compared their intakes to intact females.

It is known that large doses of EV reduce bodyweight, or retard the usual gain in weight seen among young female rats, for a number of days following injections. It is also known that doses of estradiol can induce a conditioned food avoidance and a conditioned place aversion [13,14]. To reduce the possibility that the negative events just following injections of EV will be associated with the alcoholic beverage used, we presented the alcoholic beverage 6 days following the injections.

## Results

### Experiment 1: Doses of EV and Intake of Sweetened Alcoholic (12% ethanol) Beverage

The 10 rats who received ovariectomies did not gain weight on the day following surgery, but thereafter showed the characteristic trend toward gaining more weight than their intact counterparts. All rats received their injections (EV in carrier or EV's carrier) after they were weighed on the 6^th ^day after the surgeries of ovariectomies.

**Figure 1:**
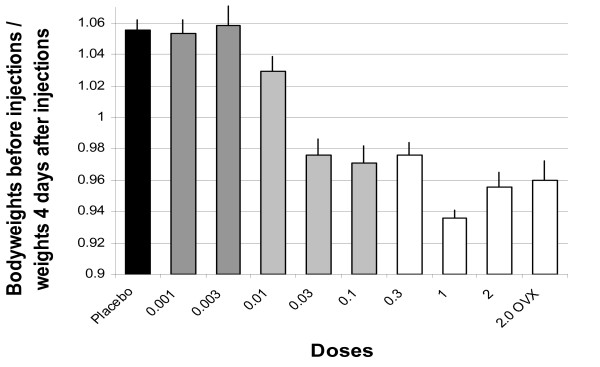
**Changes in Bodyweights with Various Doses of EV**. Mean ratios using baseline (the weight of the rats just before injections) and the mean of Day 4 after injections (after) to obtain a % change in bodyweight across doses of EV. A ratio greater than 1 indicates that the subjects gained weight and a ratio less than 1 indicates that subjects lost weight. The mean weight across all rats just before injections was 223.5 g and it was arranged so that the groups' weights were nearly the same before injections. N = 10 females a group. The lines above the bars depict standard errors of the means. The two darkest gray bars denote p-values associated with t-test comparisons to the placebo values and those values are ps > 0.75. The p-values associated with the lighter gray bars range from 0.04 to 0.01. The p-values associated with doses depicted by the white bars are ps < 0.0000001.

Figure 1 summarizes the initial effects of doses on bodyweights. As expected for healthy, young rats living in cages by themselves with food and water always available, the placebo controls gained about 1% of their bodyweight daily. Generally, the larger doses induced a loss in weight that reached a maximum at about 4 days after the injections. Subsequent to the initial loss of weight, the rats gained weight regularly. The pattern of loss and return to regular weight gain, for the doses of 1 and 2 mg/rat, are very similar to that reported in Reid et al. [5]. As expected, EV prevented the rapid weight gain associated with ovariectomies.

**Figure 2:**
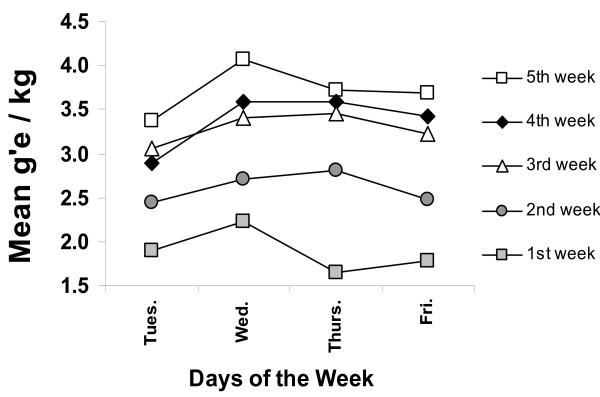
**The progression of intake of ethanol across weeks**. This figure depicts mean intake of ethanol as a function of week of presentation of the alcoholic beverage for the placebo-control group and the two groups getting the smallest doses of EV (an enlarged control group). Notice that there is a marked increase in intake from the 1^st ^week to the 2^nd ^and from the 2^nd ^to the 3^rd^. Intakes tend to stabilize across the 3^rd ^to 5^th ^week. This pattern of increases is typical of rats presented palatable alcoholic beverage. Other data [e.g., 5] indicate that, without any further intervention, intakes increase only slightly beyond those of the 5^th ^week of opportunity to take palatable alcoholic beverages.

Six days after injections, all rats were presented with sweetened alcoholic beverage for the 6 hr of initial darkness of a daily light-dark cycle. The beverage (12% ethanol, by weight) was presented on Tues., Wed., Thurs., and Fri. of each week. Figure 2 presents the progression in intake of alcoholic beverage, in terms g'E/kg, for the placebo controls and the two groups of the smallest doses (those showing no weight loss with injections). Notice that intakes were modest during the first week, but gradually increased during the next two. By the 4^th ^and 5^th ^week, intakes were substantial. If EV were to induce an increment in taking alcoholic beverage, it would have to increase already large intakes associated with this kind of regimen.

**Figure 3:**
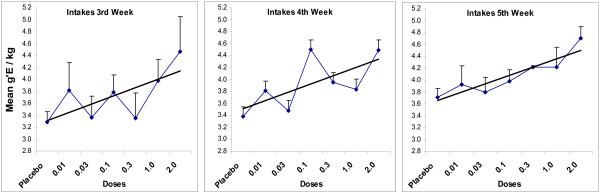
**Mean Grams of Ethanol Taken Daily during Weekly Presentation of Alcoholic Beverage**. This figure presents a summary of the effects of the various doses on intake of alcoholic beverages by presenting the mean weekly intake of the females during the 3^rd^, 4^th ^and 5^th ^week of presentation of alcoholic beverage. The data point labelled placebo is the mean of the groups getting no EV and the two groups getting the smallest doses of EV (0.001 and 0.003 mg/female). The bars are standard errors of the mean. The lines without data-points are trend lines of best fit to the data.

Figure 3 presents the salient findings with intake of ethanol across doses, excluding the ovariectomized subjects. Initial analyses, by way of ANOVA for repeated measures, indicated that all main effects (doses, the factor associated with weekly scores, and the factor associated with days within weeks) and interactions were reliable sources of variance, indicating further analyses were appropriate. With respect to gain in bodyweights (Figure 2) and data of intakes, at no comparison were scores of the placebo group and groups getting EV at 0.001 and 0.003 statistically different from one another. Consequently, these three groups were combined to obtain an enlarged control group, labeled placebo in some figures (n = 30) to further assess the effects of doses on intakes.

To further assess the effects of doses on intakes, an ANOVA for repeated measures was done using the mean intakes for each week of the first 5 weeks of presentation of alcoholic beverage using the enlarged placebo-control group and each of the groups getting larger doses. The ANOVA yields for the repeated measures of weeks F(4, 28) = 141.28, p < 0.0001, showing that intakes increase as weeks of presentation continue (this main effect is shown in Fig. 2). The values for the group effect (doses) are F(7,92) = 2.12, p = 0.05. The values associated with the interaction are F(28,368) = 1.82, p = 0.008. The ANOVA indicates it is appropriate to look for simple main effects (doses) at each week.

The one way ANOVAs for simple main effects at Weeks 1, 2 and 3 indicate that groups were not reliably different from one another. Similar ANOVAs, however, yielded values indicating significant differences among groups at Weeks 4 and 5. For Week 4, the values are F(6,83) = 3.69, p = 0.003; for Week 5, F(6,83) = 2.03, p = 0.07. These results indicate that it is appropriate to compare scores for each dose with one another. Using Fisher's test for post hoc comparisons, the scores of placebo were compared with each dose of EV for each week. With one exception, only the scores associated with the 2 mg dose meet standards for being statistically significant (ps < 0.05), but notice that the trends indicate a dose-response relationship. It may be, as indicated by the significant value associated with the 0.1 mg EV-value (middle panel of Figure 3), that the largest effect on intake for any given dose may only be at certain times and those effects may wane across time.

**Figure 4:**
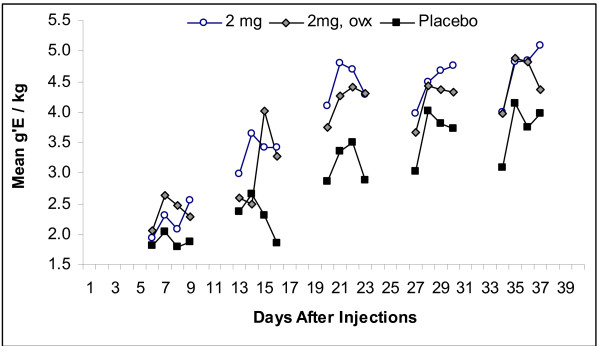
**Mean Grams of Ethanol Taken for Each Day of Presentation of 12% Ethanol Solution for Three Groups of Subjects**. The mean intakes for each day of presentation of alcoholic beverage for three groups of subjects, a placebo-control group and two groups that had received 2 mg of estradiol valerate (n = 10 a group), one of which had ovariectomies. Notice that there is considerable variation associated with days within weeks. More importantly, notice that the intakes of the two groups receiving 2 mg of EV drank more during the 3^rd^, 4^th^, and 5^th ^week of opportunity to do so.

Figure 4 serves two purposes. Analyses of the intake data (some of which are depicted in Figure 3) indicated that there were statistically significant differences among days within weekly blocks. To depict the kind of scores leading to those statistically significant effects, the scores for days within weeks are presented for some of the groups (Figure 4). Although the change in scores following periods of no alcoholic beverage (withdrawal) to those when alcohol is again provided are of interest to theory concerning the relevance of those changes to relapse to drinking large amounts of alcohol (the so-called alcohol deprivation effect), the interest here is only on estradiol's potential effects. Further analyses of the effects associated with the factor of days are not presented here. The effects of ovariectomies are also summarized in Figure 4.

Did ovariectomies modify the effects of EV, 2 mg? To address that, we compared intakes of three groups: (a) intact females who received 2 mg of EV, (b) females who received both ovariectomies and 2 mg of EV, and (c) the enlarged placebo group (placebo, 0.001 and 0.003 doses of EV) using weekly means for each 5 weeks of alcohol availability. The data-set corresponds to a 3 by 5 ANOVA for repeated measures with factors for the three groups of subjects and 5 weeks of intakes. An ANOVA of the data associated with the factor of weeks of testing yields an F(4,8) = 50.57, p < 0.0001, indicating again (Figure 2) that intakes progress across weeks of opportunity to drink. The values from the ANOVA for the group effect are F(2,47) = 5.02, p = 0.01. The interaction was not a reliable source of variance. The statistically significant group effect indicates that further comparisons among groups are appropriate.

Using post hoc adjustment of critical differences for multiple comparisons, further analysis indicated that the intact females getting 2 mg of EV differed from the controls (p = 0.008), as expected from the previous presentation of the findings. The analysis indicated that the group with ovariectomies getting 2 mg of EV drank more alcoholic beverage than controls (p = 0.03). There was no indication that the two groups getting EV differed significantly from one another (p = 0.65). In brief, the analysis supports the conclusion that EV, 2 mg, enhances intakes of alcoholic beverage in both intact and ovariectomized females.

**Figure 5:**
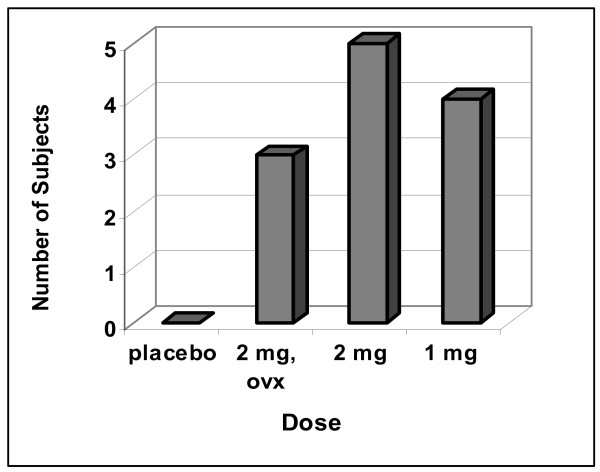
**Number of Subjects taking more than 80 g'E/kg**. This graph is another way of characterizing the effects of the larger doses of EV. It depicts the number of subjects taking more than 80 g'E/kg across the 5 weeks of opportunity to drink (i.e., 20 days of opportunity). 80 g'E/kg is larger than the upper limit of the range of the placebo group. Notice that 50% of the group getting 2 mg EV drank more than the most avid drinker of the placebo group.

Figure 5 is another way of characterizing the effects of large doses of EV. It is a tally of the number of subjects taking more ethanol than the placebo-control subject that took the most. Five females of the 2 mg dose of EV drank more than 80 g'E/kg during the 20 days of opportunity to do so, i.e., more than 80 g'E/kg during the 120 hr of opportunity to drink; more than 0.6 g'E/kg an hour.

### Experiment 2: Presentation of 18% Ethanol in Sugar Water to Rats Given High Doses of EV and Placebo Controls

We have assessed the effects of EV using a variety of kinds of alcoholic beverages. We have not, however, used a beverage with a concentration more than 12% ethanol (weight to weight). To see what would happen when a concentration of 18% was presented, we continued to assess intakes of sweetened alcoholic beverage using four groups of Experiment 1: (a) the group of 2 mg of EV, (b) of 2 mg with ovariectomies, (c) of 1 mg of EV, and (d) placebo-controls. After the 6 weeks of the weekly regimen of Experiment 1, the subjects continued testing with the only change being that the alcoholic beverage was 18% rather than 12%.

**Figure 6:**
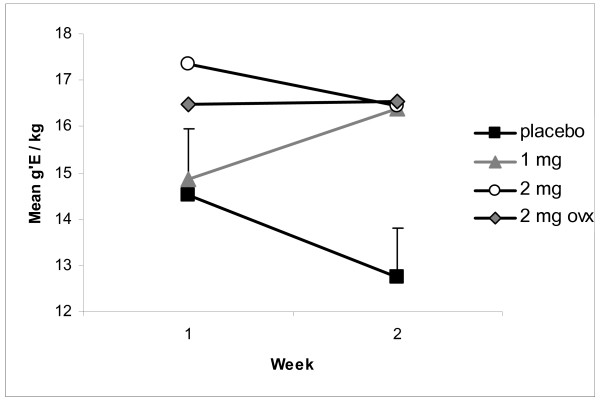
**Weekly Ethanol Intake after Introduction of 18% Ethanol Solution, Weeks 7 and 8 following Introduction of Opportunity to Drink Alcoholic Beverage**. The figure summarizes intakes during the first two weeks of presentation of 18% ethanol solution. The values are the means of the total intake during the four days of each week of the procedure (n = 10/group). The first day of the 18% beverage was 57 days after the single injection of EV. Notice that the group of placebos reduced their intakes during Week 2, but that EV-treated groups did not. Comparisons using t-tests of EV-treated groups compared to placebo group indicates that no group was statistically different than placebo controls (ps > 0.20). One subject of placebo controls drank more ethanol during Week 1 than it had previously and all other subjects. At week 2 of the figure, t-tests indicate that each group was reliably different from placebo controls, all ps < 0.04.

Figure 6 summarizes the results of introducing the more concentrated ethanol solution. During the first week of exposure to the 18% beverage, all four groups took large amounts of ethanol. During the 2^nd ^week, however, the placebo controls reduced their intakes while the subjects of EV did not. The high levels of intake of the 2 mg EV group occurred more than 50 days after the single injection of EV. The ovariectomized females drank more, on average, than the placebo controls on every day of testing.

### Experiment 3: Measures of Circulating Estradiol and Autopsy Results

At the end of measures of ethanol intake (Experiment 2), we took vaginal smears daily for 12 days from the females of 2 mg EV (only intact), 1 mg EV and placebo controls. After histological inspection of the cells of those smears, the idea was to determine what day during the estrous cycle the females were on when we sacrificed them while taking samples of blood. The idea was to be able to compare the circulating estradiol values to known standards (which, of course, vary across the days of the cycle) as well as the values among the groups.

On the day of the last vaginal smear, the females were sacrificed and trunk blood taken to measure circulating levels of estradiol. When we sacrificed the females, we also inspected the area of the ovaries of the subjects given ovariectomies.

The autopsy indicated that there were no signs of ovaries in nine of the ten females that received surgeries. One of the ten had a very small remnant of ovary on one side which was so small that we included this subject in all analyses of the data. These observations, plus the low level of circulating estradiol (mean = 10.4 pg/ml), indicate that the surgeries accomplished the goal of removing the ovaries.

After inspection of the vaginal smears, the conclusion was reached that the females given EV (1 or 2 mg) and placebos and exposed to a lengthy regimen of intake of alcohol were not cycling, at least, not regularly or in any discernible pattern. Furthermore, as can be seen from an inspection of means and the large standard deviations of circulating estradiol (Table 1), there was no statistically significant difference among the groups despite the fact that some had ovariectomies and some had only placebos. Evidently, the regimen of alcohol availability and the intake of large amounts of alcoholic beverage, when available, had a profound effect on the production of estradiol by the intact females.

**Table 1:** Estradiol levels

Group	Placebo	1 mg EV	2 mg EV	2 mg EV, OVX
Mean (pg/ml)	18.9	12.9	17.6	10.4
Standard Deviation	37.2	15.2	14.5	6.9

Note: The large standard deviation of the placebo group is due to one extra large value (122 pg/ml).

### Experiment 4: Intakes immediately following 2 mg EV injections

When rats have had many days to take sweetened alcoholic beverages, they gradually increase their intakes over about 3 weeks and then their intakes tend to stabilize (placebo-values depicted in Figure 2 and 4). In a group of rats that had stable, high levels of intakes of saccharin sweetened alcoholic beverage, 2 mg of EV markedly reduced intakes. Intakes gradually increased and eventually became more than before injections and more than those given placebos [5]. EV, 2 mg/rat, reduced intakes of a saccharin solution (no ethanol), but produced less reduction in intake of a sugar solution [11]. This experiment asked whether the 2 mg EV also reduced the steady intake of females taking a sugar sweetened alcoholic beverage.

The subjects were those who had received 0.001 and 0.003 mg of EV in Experiment 1. At the end of the first 5 weeks of opportunity to drink, they were drinking nearly the same amount as placebo controls. They were then randomly assigned to get either 2 mg of EV or placebos.

**Figure 7:**
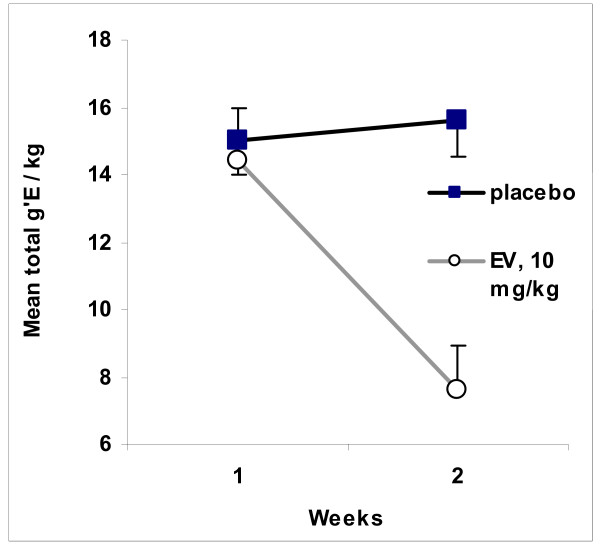
**Intake of Alcoholic Beverage Immediately Following 2 mg of EV or Placebo**. This figure summarizes the change in intakes of two groups of females. Both had been taking alcoholic beverage for 4 days a week for 4 weeks before the start of this experiment. They continued that regimen for another week (Week 1 here, a baseline measure). After Week 1, one group received placebos. The other group received 10 mg/kg of EV. The data are means of total intake taken during the week (across 4 days). The bars represent standard errors of the mean. As can be seen by the overlap of error bars, the groups did not differ significantly at Week 1 (baseline). The group of placebo continued to take substantial amounts of ethanol and showed no significant change in intakes from Week 1 to 2, p-value for t-test for dependent groups = 0.55. As can be seen from the lack of overlap of error bars, the groups differed significantly in intake during Week 2, p = 0.0005. The dose of EV reduced intakes, p-value for t-test for dependent means = 0.003 (Week 1 vs. Week 2, EV group).

The subjects that received EV lost weight in nearly the same amount and pattern as those of 2 mg, Experiment 1. The results, g'E/kg, are depicted in Figure 7. As can be seen, the group getting EV markedly reduced their intake during the week just following their injections. These results are similar to those obtained previously with females taking other alcoholic beverages [5].

### Experiment 5: Estradiol Levels Following Doses of EV

We used 85 females of nearly the same age, weight, and strain as used in Experiment 1 to 4 to obtain data concerning the levels of circulating estradiol following the single injections of EV. These females lived for 27 days, or more, under the same conditions (e.g., food and water always available, housed individually) in the same room as the females of Experiments 1 to 4. Just after the trunk blood was taken, persons unfamiliar with the subjects' group membership inspected the nipples of the females judging their size as small, medium or large and the number of nipples meeting each judgement.

In order to have data applicable to more than this report, we gave doses in terms of mg/kg. The 10 mg/kg is similar to the 2.0 mg/rat used in other studies (and Experiments 1–4). The other doses were 7.0 and 3.0 mg/kg (comparable to log units). Samples were taken 4, 12, and 27 days after injections. Samples were taken from placebo-treated subjects 27 days after their injections.

**Figure 8:**
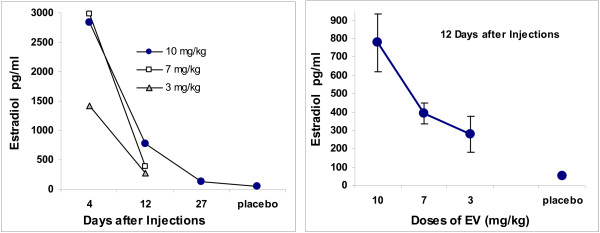
**Estradiol Levels Following a Single Injection of One of Three Doses of Estradiol Valerate**. Serum levels of estradiol following single injections of 10.0, 7.0, or 3.0 mg/kg of estradiol valerate. Samples were taken at 4, 12 or 27 days after injections of EV. Placebo samples were taken at 27 days after injection of carrier of EV. The 10.0 mg/kg dose is very similar to the 2.0 mg/female dose given to subjects of Experiment 1. The right panel is an expansion of the data taken 12 days after injections. The error bars are standard errors of the mean; the standard error of the mean for placebo scores = 5.1.

The results are summarized in Figure 8. It is clear, and as expected, the level of circulating estradiol is a function of size of dose and time after dosing. The levels achieved after the largest doses are massive in comparison to doses that clearly modify the estrous cycle and are large in comparison to doses achieved during pregnancy.

None of the placebo-treated rats had enlarged nipples. When judgements were made on those inspected at 4 days after injections of EV, none of the females were judged to have enlarged nipples. When judgements were made of those of 12 and 27 days after injections of EV (regardless of dose), all rats were judged to have multiple medium and large nipples. With no overlap in distributions, there is statistical significance between placebo-treated and EV-treated groups and among times of injections for EV-treated subjects.

## Discussion

A potential criticism of these findings is that, by providing sugar sweetened alcoholic beverage as the alcoholic beverage to be assessed, we have confounded taste and calories with the unique properties of ethanol that might sustain high levels of intake. This is a cogent criticism, if the goal of the research was to assess the effects of EV on intake of ethanol itself rather than the propensity to drink a palatable alcoholic beverage. If one is to assess the drinking of an alcoholic beverage, there is no way to separate taste and calories from a beverage containing ethanol. Alcoholic beverages of water and ethanol have both taste (probably bitterness covarying with concentration of ethanol) and calories. The goal here was to assess the effects of doses of EV on intake of an alcoholic beverage taken in large amounts. If we had provided our subjects with merely ethanol and water, the rats would not have taken sufficient beverage to achieve considerable blood ethanol levels. By using a palatable alcoholic beverage, we are more apt to measure the effects of ethanol because taste and calories are not limiting the intakes. With the beverage used here, it is the ethanol that is limiting intakes (as demonstrated by the gradual increase in intakes across weeks) (Figure 2) and is the salient feature of the beverage. Realizing that these arguments have not convinced those who study rats' intake of alcoholic beverage to use highly palatable beverages, we [5] demonstrated that EV enhanced intakes of alcoholic beverages when the beverage is only ethanol and water (of course, intakes of ethanol were much smaller, but the smaller amounts were enhanced).

The problematic consequences of drinking alcoholic beverages are directly related to the amount of ethanol consumed regardless of the flavour of the beverage. The beverage used here to some human's taste has a clear taste of ethanol (it is more than 24% proof) and tastes like after dinner drinks or fortified wines. Similar drinks are readily available to women who have been given doses of estrogenic medicines.

In the group getting 2 mg of EV, there was one subject that did not drink any alcoholic beverage for a week. It showed what some have called a neophobia. Another female did not show the characteristic weight loss associated with injection of EV (probably some inadvertent failure of the injection) and its intake was similar to placebo controls. These two subjects' data are included in the data presented. If their data were not included, the effects of 2 mg of EV would appear somewhat larger and more consistent. The possibility of neophobia can be controlled for by presenting a to-be-tested ingesta before injections for sufficiently long period for all subjects to be taking it.

The initial effects of continuous estradiol are a loss of bodyweight (Figure 1) and a reduction in intake of alcohol (Figure 7) and other ingesta [12,23,24]. If the loss of bodyweight signifies a malaise, as other data indicates [13,14], these initial effects, when paired with discernible ingesta by way of well-known conditioning mechanisms might mask the development of what otherwise might be an enhanced appetite for alcohol or other ingesta. With the recognition that we might be establishing a conditioned taste aversion, if we paired the initial effects of our injections with the first presentations of alcoholic beverage, we waited 6 days after injections to introduce the rats to the alcoholic beverage.

It is easy to see, in retrospect, how the usual procedures for assessing a drug on intake of alcohol, when used while assessing estradiol, would have produced results leading to the conclusion that estradiol did not enhance intakes. Special procedures are needed to observe estradiol's potential to enhance intake of alcoholic beverages (i.e., those involving not pairing the initial effects of estradiol with initial presentations of the beverage and prolonged testing allowing sufficient time to have the rats develop their taste for alcoholic beverage both before and after application of continuously high levels of estradiol). When those special procedures are used, continuous application of estradiol can induce an enlarged appetite for alcoholic beverages. The results of the relatively few studies that have assessed the effects of injections of estradiol on intake of alcoholic beverages lead to the conclusion that the initial effects of estradiol and the continuous effects are different.

In this experiment, although we did separate the initial effects of EV from presentation of alcoholic beverage, we may not have had ideal conditions for seeing the effects of small doses of EV. The major effects of small doses may have faded during the time the females were developing their taste for alcohol. If the smaller doses' effects waned while the rats were acquiring a taste for the alcoholic beverage, we may have limited the opportunity to see marked enhancing effects with small doses. Using a regimen for establishment of stable intakes before injections and then not pairing the initial effects of EV with the to-be-tested food, Boswell et al. [28] has shown that small doses of EV (0.19 mg/kg) enhance intakes of chocolate cake mix batter when care is taken to control for the initial effects of EV.

It has been known for some time that opioid receptor antagonists reduced and opioid receptor agonists enhanced intake of alcoholic beverages and other ingesta [25]. Juárez et al. [26] have hypothesized that the initial effects of estradiol are to downregulate opioid receptors inducing a state similar to administering an opioid antagonist. Following downregulation, it was hypothesized that a rebound upregulation would induce the effects of opioid agonists. The idea emerges that the initial effects of continuous estradiol is one that may reflect a malaise and a down regulation of opioid receptors producing conditions for reduced intake and potential for conditioned taste avoidances to novel ingesta. Given that small doses of morphine (smaller than those inducing analgesia) will enhance intakes of alcoholic beverages, and do so when given day after day [27], it is reasonable to hypothesize that events that might enhance opioidergic tone would increase intakes. The estrogenic effects may be susceptible to manipulation by opioidergic drugs during the initial effects of estradiol; for example, small doses of morphine may prevent the longer term upregulation of receptors.

Pregnancy is characterized by initiation of prolonged, high levels of estradiol and is often associated with a finicky approach toward food followed by enhanced appetite [11]. The presence of enlarged nipples in the females following large doses of EV indicate that the continuous estradiol made changes that are associated with pregnancy.

Based on one perspective, the conclusion to be reached from our dose response study is that only very large doses of EV enhanced intakes of alcohol (with one exception, only the doses of 1 and 2 mg produced scores that were statistically different than placebo scores). Such a perspective would push one toward explanations of the effects of large doses EV in terms of its reputed toxic effects on the arcuate nucleus of the hypothalamus. If the effect is based in toxicity of very large doses, maybe the effect is not germane to conditions of women and their intake of estrogenic medicines. From another perspective, however, Juárez et al. [26] recently showed that smaller doses of estradiol did enhance intakes when opportunities to drink were not associated with the initial effects of EV. Also, the dose-orderly effects associated with Figure 3 suggest that under other circumstances of testing smaller doses might enhance intakes. The data support the hypothesis that the enduring effects from a single injection of EV are related to doses similar in magnitude of 1 and 2 mg/female. Smaller doses may produce transitory effects.

There are data indicating that women with higher levels of circulating estradiol drink more than their counterparts [29,30]. Prior to a small number of some recent studies [5,9,10,31], including this one, the consensus from laboratory-studies was that estradiol reduced intake of alcoholic beverages [6,7] and ingesta in general [12,23,24]. Conclusions such as a surfeit of estradiol might enhance appetite for alcohol that might be derived from the correlations obtained from the epidemiological studies of women [29,30] were not supported by studies from the experimental laboratory where estradiol was administered and intakes of alcohol measured. The newer studies from the laboratory do indicate that there are circumstances under which estradiol can enhance intakes of alcoholic beverages and open the possibility that estrogenic stimulation may enhance appetite for alcohol. A sustained increased appetite for alcohol, of course, is the condition of AAA.

When we measured estradiol levels 67 days after injections, the estradiol levels were very low, so low they were similar to ovariectomized females. The conclusion that continuous application of estradiol can induce marked increases in intake of alcoholic beverage seems contrary to the finding that toward the end of our testing, subjects given large doses of EV had estradiol levels that were small, but intakes of alcohol were large. In other experiments [9,10], about a 100 days after injections, EV-treated groups were drinking more than the placebo-treated. The resolution of the apparent conflict, perhaps, resides with the idea that 2 mg of EV induces supraphysiological levels of estradiol that eventually lead to disruption of the arcuate nucleus [2]. The idea is that the continuance of high levels of estradiol, in the relatively short run, and disruption of the arcuate nucleus due to prolonged high levels of estradiol are both conditions of high intake of alcoholic beverages.

The mean level of circulating estradiol of the placebo-treated subjects that had no access to alcohol is 53.2 pg/ml (standard error of mean = 5.1) compared to the placebo-treated subjects that were on the regimen of alcohol presentation whose mean is 18.9 (standard error of mean = 9.3) (p-value associated with a t-test for independent samples = 0.003) (see Table 1 for other values). The measures of circulating estradiol were conducted in exactly the same way, but the females of the alcohol regimen were somewhat older and had lived a shorter time in individual cages. Nevertheless, the difference in estradiol levels is large and suggests that periodic intake of large amounts of alcoholic beverage might disrupt production of estradiol in females.

Large doses of EV (2 mg/rat or 10 mg/kg) can provide continuous, supraphysiological levels of circulating estradiol for an extended period. To achieve that, however, very large levels of circulating estradiol are seen initially. EV surely does not provide constant amounts of estradiol across a period after injection. The long half-life of EV indicates that with multiple doses of EV, there could be an accumulation of estradiol. Two mg doses of EV along with a progesterone-like drug, given monthly, are the doses used in birth control drugs touted as effective for use by women of underdeveloped countries with special reference to Latin America [32].

## Conclusion

These data confirm that a single injection of EV reduces rate of weight gain in female rats in a dose-orderly way, with larger doses leading to loss of weight. The disruption in weight gain is temporary lasting only a matter of days even following very large doses of EV (e.g., 2.0 mg/female). There is tolerance to the anorexic effect of estradiol as manifest in a return to ordinary rate of weight gain.

These data confirm that an injection of 2.0 mg of EV can reduce rats' intake of alcoholic beverage when the alcoholic beverage is presented during the days immediately following injections even when that intake is primed by presenting sugar sweetened beverage for a number of days before injections.

These data confirm that a single injection of 2 mg of EV/female rat can also induce an enhanced appetite for alcoholic beverage. The effect can occur with a variety of alcoholic beverages including 12 and 18% ethanol solutions sweetened with sugar.

The 2 mg dose of EV can enhance intakes in females without ovaries. This restricts some theories of how EV might induce its effects.

The very large doses of EV induce correspondingly very high levels of estradiol that gradually wane with time. At 27 days after dosing, the levels of circulating estradiol are still at levels ordinarily achieved during pregnancy.

A regimen of inducing periodic large intakes of ethanol disrupts female rats' production of estradiol in placebo controls.

The data from the relatively small number of studies administering estradiol and measuring intake of alcoholic beverages [5,6,9,10,26,31,33-35], using rodents, are concordant with one another, i.e., all studies show similar effects. Shortly after injection of a preparation such as EV or with continuation of dosing by way of multiple injections of estradiol benzoate, there are reductions in intakes of alcohol. If, however, the estradiol levels are maintained at a high level for a number of days, intakes gradually increase to levels before injection. Further, there are circumstances under which intakes not only return to baseline levels but intakes increase to very high levels, higher than preinjection levels and higher than placebo controls. These generalizations are apparently not dependent on strain of rats, whether the females are intact or ovariectomized, kind of alcoholic beverage presented during the testing, or the particular laboratory (and related small procedural differences) in which the tests were made.

Many circumstances will cause rats to reduce intake of ethanol, e.g., anything inducing a malaise. Only a few circumstances reliably induce significant increases in already high levels of intake of ethanol, particularly long-lasting enhanced intakes. Consequently, the findings that estradiol can increase intakes might be of interest to those building theory of alcohol use disorders.

Epidemiological studies[29,30] have shown that women with high levels of circulating estradiol drink more alcohol than their counterparts. These epidemiological results clashed with the early findings from the experimental laboratory showing that administration of estradiol reduced intakes of alcohol. The later studies from the experimental laboratory have now demonstrated that there are circumstances under which estradiol can enhance appetite for alcohol. All of the results converge to support the idea that the interrelationship between estrogenic processes and appetite for alcohol should be explored more thoroughly. This is not only the case because alcohol use disorders among women are increasing [36], but also because studies of women show relationships between estrogen levels, alcoholic intake and breast cancer [37,38].

## Methods

### Subjects

The subjects of Experiment 1 to 4 were 100 female rats from the colony maintained by the *Instituto de Neurobiología*, UNAM Campus, Juriquilla, Querétero, México. The rats were derived from the Wistar strain. Upon arrival at the room of the experiment, they were housed individually in cages with wood chips on the floor. The room of the cages was maintained at 21°C and had artificial lighting. The lights came on at 10 pm and went off at 10 am. The lights were turned on, briefly, at 4 pm, to facilitate removal of the bottles containing alcoholic beverage from the rats' cages. The rats always had food and water available.

The 86 subjects of Experiment 5 were as similar to those of Experiments 1 to 4 as we could manage. They were of the same sex, strain, and nearly the same weight when they were placed in the room in which Experiments 1 to 4 was conducted. The methods and care of the animals were approved by the institutional care committees of each institution with which the authors were affiliated.

### Doses of EV and Ethanol Solution

EV was purchased from Sigma-Aldrich (St. Louis, MO, USA). The carrier of EV was sesame seed oil (Sigma-Aldrich). All injections were 0.2 ml, given intramuscularly. One group, the placebo controls (n = 10), received only 0.2 ml of oil. Eight other groups (n = 10 each) received one of eight doses of EV: 0.001, 0.003, 0.01, 0.03, 0.1, 0.3, 1.0 or 2.0 mg/rat. The 2.0 mg dose was the most characteristic dose used in the experiments assessing EV's effects on alcohol intake. The dose of 1.0 mg was used in one experiment and seemed to be effective. The other doses are graded fractions (in terms of log doses) of the 1 mg dose.

The history of the research dictated the choice of doses, i.e., we used the dose that was supposedly toxic to the neurons of the arcuate nucleus, i.e., 2 mg/rat [2-4,22]. Notice, however, that the doses are not precise. The subjects' weights varied; consequently some subjects received more EV in terms of mg/kg than others. Also, the rats' weights change across the period during which estradiol is being released. In Experiment 5, we gave doses in terms of mg/kg, i.e., 10, 7 and 3 mg/kg. The 2 mg/female dose used in most experiments is very similar to 10 mg/kg (i.e., about 200 g is the modal weight of subjects)

The alcoholic beverage was a sucrose-sweetened ethanol solution. For each 100 g of beverage, there were 12 g of absolute ethanol (99.8% ethanol), 5 g of sucrose (table sugar from a local market) and 83 g of water. This beverage had been used in other experiments (e.g., [25]) with male rats. Based on those findings, it was expected that initially the placebo-controls would take only small amounts of the beverage, but increase intakes until they were taking substantial amounts. In one experiment, an 18% ethanol solution was used.

### Procedure, Experiment 1

After a few days to habituate to individual caging, the rats were weighed daily. Ten were selected randomly to receive ovariectomies. After determining weights of all subjects across 5 days post surgery, the remaining 90 were assigned to groups so that the mean weights of the groups were similar and each group contained 10 subjects. After they were weighed on the 6^th ^day (mean weight = 223.5 g), all rats received an injection. Doses were randomly assigned to groups. The 90 rats received one of nine injections: 0.0 (placebo), 0.001, 0.003, 0.01, 0.03, 0.1, 0.3, 1.0, or 2.0 mg of EV. Injections were intramuscular in the hip. The rats of ovariectomies received 2.0 mg.

Six days after injections, all rats were presented with the sweetened alcoholic beverage from 10 am to 4 pm (initial hours of darkness). The alcoholic beverage was presented on Tues., Wed., Thurs., and Fri. of each week. For two groups (those of 0.001 and 0.003), the procedures changed at the end of the 5^th ^week (Experiment 3). Testing was terminated for the groups of 0.03 to 0.3 at the end of 5 weeks of testing. The groups of 2.0 (both intact and those with ovariectomies), 1.0 mg/kg, and placebos continued for another week as before, i.e., for a 6^th ^week. Then, the procedure was changed for the four groups; they were given 18% ethanol as their alcoholic beverage for additional 2 weeks of the regimen (Experiment 2).

### Procedure, Experiment 2

The weekly regimen of Experiment 1 was continued for 4 groups of subjects just as before except that the alcoholic beverage that was presented was 18% ethanol (by weight) rather than 12%. The four groups were those of 2.0 mg of EV (intact and those with ovariectomies), 1.0 mg of EV and the placebo controls (0.0 mg of EV). The new alcoholic beverage was 18 g absolute ethanol, 5 g sucrose and 77 g of water for each 100 g of solution.

### Procedure, Experiment 3

At the end of Experiment 2, no further alcoholic beverage was presented. Beginning week 9 following injections, a sample of loose cells from the vaginal wall was taken every day for 12 days during the first hour of dark and placed on glass slides. The cells were later stained and inspected microscopically.

After the last acquisition of cells from the vagina, all subjects were sacrificed by decapitation and their trunk-blood taken for assessment of estradiol levels (see below). With the taking of blood, an autopsy of the rats with ovariectomies was performed with the goal of determining if the ovaries had, indeed, been removed.

### Procedure, Experiment 4

The subjects of this procedure are the subjects of Experiment 1 that received either 0.001 or 0.003 mg of EV. Neither dose had a reliable effect on rate of gain in bodyweight or intake of alcoholic beverage. At end of the 5^th ^week of access to alcoholic beverage, one group was randomly selected to receive placebos (carrier of EV) and the other 10.0 mg/kg of EV. Group membership was determined, in part, so that each group had the same number of subjects of the former group and had similar intakes of ethanol and bodyweights. The group to receive EV was determined by the flip of a coin. Injections occurred on Friday of the 5^th ^set of weekly presentations of alcoholic beverage and about 2 hr after their usual 6-hr opportunity to take alcoholic beverage. The schedule of presentations of alcoholic beverage remained the same as programmed during the previous 5 weeks for another week (the week after injections).

### Measures of Estradiol in Serum

The samples of trunk blood were from subjects of Experiments 2 and 86 additional subjects. The samples were allowed to coagulate, thereby providing an initial separation of plasma from cells. Then, a sample of plasma was taken and submitted to centrifugation to further separate dense material from plasma. The plasma was then frozen and stored at -70°C until assayed for estradiol.

The samples were gradually thawed, e.g., keeping them at -5°C for 15 minutes then at 4°C for 15 more minutes. The analysis was done at room temperature. The analyses were done by use of IMMULITE chemiluminescent counter using IMMULITE Estradiol kit from Diagnostic Products Corporation (Los Angeles, CA). This kit has high specificity for the target hormone and low cross-reactivity with other steroids. IMMULITE Estradiol has 100% specificity for E_2_. Cross-reactivity is 0.535% with estriol; 2.09% with estrone and testosterone is undetectable. Sensitivity goes from 20 to 2000 pg/ml.

### Data-reductions and Statistics

The bottles (equipped with ball point sipping tubes) were weighed before and after their daily presentations. The differences in weights, corrected for spillage, are the raw data for ingestion of alcoholic beverage. The amount to correct for spillage was determined by placing four or more bottles on empty cages and taking the mean amount lost across the measurement period as a correction factor. The usual amount was less than 1 ml and varied somewhat across days but little across bottles. The rats were weighed just before presentation of the beverage. Using these measures, g'E/kg were calculated.

The experimental design for Experiments 1 to 4 conforms to the factorial design for an analysis of variance (ANOVA) for repeated measures having factors associated with the groups of subjects (doses) and periodic measurements of ethanol intake (g'E/kg). When reliable effects emerged, tests for simple main effects were done. When there were opportunities for multiple comparisons of groups' performances, a correction for multiple comparisons was used. When comparing some groups' scores, there was no need for correction for multiple comparisons, t-tests were used.

## Authors' contributions

This experiment emerged following discussions among LDR, GLQ, MLR, and RAP. The other authors joined the discussions as the data were collected and drafts (initial draft by LDR) of the report were prepared. GLQ helped prepare the final draft with collaboration of all authors. All authors were involved in most aspects of the data-collection except MAS whose role, in terms of data-collection, was limited to measures of estradiol in serum.
